# Characterization of protein *N*-glycosylation by tandem mass spectrometry using complementary fragmentation techniques

**DOI:** 10.3389/fpls.2015.00674

**Published:** 2015-08-28

**Authors:** Kristina L. Ford, Wei Zeng, Joshua L. Heazlewood, Antony Bacic

**Affiliations:** ^1^ARC Centre of Excellence in Plant Cell Walls, School of BioSciences, The University of MelbourneMelbourne, VIC, Australia; ^2^Physical Biosciences Division, Joint BioEnergy Institute, Lawrence Berkeley National LaboratoryBerkeley, CA, USA

**Keywords:** glycosylation, fragmentation, electron-transfer dissociation, post-translational modification, tandem mass spectrometry

## Abstract

The analysis of post-translational modifications (PTMs) by proteomics is regarded as a technically challenging undertaking. While in recent years approaches to examine and quantify protein phosphorylation have greatly improved, the analysis of many protein modifications, such as glycosylation, are still regarded as problematic. Limitations in the standard proteomics workflow, such as use of suboptimal peptide fragmentation methods, can significantly prevent the identification of glycopeptides. The current generation of tandem mass spectrometers has made available a variety of fragmentation options, many of which are becoming standard features on these instruments. We have used three common fragmentation techniques, namely CID, HCD, and ETD, to analyze a glycopeptide and highlight how an integrated fragmentation approach can be used to identify the modified residue and characterize the *N*-glycan on a peptide.

The identification, characterization and quantification of post-translational modifications (PTMs) in proteins are a major challenge in the field of proteomics (Heazlewood, 2011). Protein glycosylation is one of the most commonly occurring PTMs with estimates of around 50% of the cellular proteome predicted to be glycosylated (Van den Steen et al., 1998). As well as being a commonly occurring PTM, protein glycosylation is one of the more difficult protein modifications to investigate due to the heterogeneity of the glycan structure (Song et al., 2011). Indeed, the examination of protein glycosylation is further complicated by the variations in the glycan structure (glycoforms) that can occur on a given polypeptide (Rudd and Dwek, 1997). Aberrations in protein glycosylation result in severe developmental abnormalities in diverse species including mammals (Furuichi et al., 2009) and plants (Lerouxel et al., 2005). Protein glycosylation primarily occurs in the secretory system (ER and Golgi) and can result in functional changes, influence subcellular localization, and protein stability (Oxley et al., 2004; Zhou et al., 2005). Identifying and profiling protein glycosylation is thus essential to define the underlying subtleties of a complex proteome.

The application of tandem mass spectrometry (MS^n^) as a technique for the characterization of protein glycosylation has occurred for decades (Reddy et al., 1988). However, it has been the development of proteomics-based MS that has enabled the high-throughput analyses of protein glycosylation (glycoproteomics) to occur (Morelle and Michalski, 2007; Zhou et al., 2007; Zielinska et al., 2012). To secure peptide sequences, these high-throughput approaches usually require the enzymatic removal of the glycan prior to analysis by MS (Zhang and Aebersold, 2006), thus losing information on the glycan structure and the specific site of glycosylation. The removal of the glycan is necessary since commonly applied fragmentation techniques produce spectra yielding information from the carbohydrate structure rather than the peptide backbone. This is generally due to the labile nature of the glycan moiety (Ruiz-May et al., 2012b; Saba et al., 2012) and without removal of the glycan, it is difficult to match resultant fragmentation spectra using a standard data analysis workflow. As a result, these types of approaches have thus far dominated the area of glycoproteomics.

As with other eukaryotes, plants contain an array of glycoproteins that are mainly produced in the secretory pathway and contain both *N*- and/or *O*-linked glycan structures (Ruiz-May et al., 2012a; Strasser, 2014). A number of targeted plant glycosylation studies have revealed the importance of these motifs to protein function, including enzyme activity (Kimura et al., 1999), thermal stability and folding (Lige et al., 2001), and protein solubility (Welinder and Tams, 1995). However, the prevalence of *O*-linked glycosylation and the diversity of *N*-glycan structures found in plant glycoproteins poses distinct challenges for their global analysis in plants (Gomord et al., 2010). The range of *N*-glycans identified on plant proteins are separated into three broad types; each type represents a variety of structural permutations. The high mannose type comprises structures of GlcNAc_2_Man_*5–9*_ and they mainly occur in the ER and represent immature or precursors of the complex *N*-glycan structures. The complex type of plant *N*-glycan structures represents the mature form found in the late Golgi and on extracellular proteins and have been described as comprising GlcNAc_4_Xyl_1_Fuc_1_Man_3_ to GlcNAc_4_Xyl_1_Fuc_3_Man_3_Gal_2_. Lastly, the paucimannose type represents the processing of terminal residues (GlcNAc/Fuc) from complex *N*-glycan structures resulting in a processed *N*-glycan comprising GlcNAc_2_Xyl_1_Fuc_1_Man_3_; these structures are thought to be present in vacuolar localized proteins (Rayon et al., 1998). Detailed knowledge about *O*-linked glycan structures found on plant proteins is still limited. The most widely studied proteins with *O*-linked glycans structures in plants are the extensins and arabinogalactan proteins (AGPs), which belong to the hydroxyproline-rich glycoprotein (HRGP) superfamily, where the *O*-glycan is mainly attached to modified Pro residues (hydroxyproline). The extensins are glycosylated on a series of adjacent hydroxyprolines with Ara_4−1_ followed by an *O*-linked Gal on a Ser residue (Gomord et al., 2010). In contrast, the AGPs contain large branched Gal structures also *O*-linked through hydroxyprolines that contain terminal Ara but can also include Fuc, GlcA, Rha (Nguema-Ona et al., 2014). The complexity and diversity of *O*-glycan structures and resulting glycopeptides derived from these *O*-linked glycoproteins has resulted in few MS-based studies; indeed their characterization usually requires a multifaceted approach (Hijazi et al., 2012). Consequently, we intend to address the role of complementary fragmentation techniques by MS for the characterization of *N*-glycans.

The distinct structural feature found in complex plant *N*-glycans e.g., the presence of an α-1,3-linked fucose in *N*-glycans (Tretter et al., 1991), has resulted in few glycoproteomic studies as the established workflows require some adaptation (Song et al., 2013). Consequently, the first attempt at applying the emerging glycoproteomic technologies to plant material was only undertaken a few years ago (Zielinska et al., 2012). Prior to this, some plant glycoproteomics had been conducted including in Arabidopsis (Minic et al., 2007) and tomato (Catalá et al., 2011), however these studies only characterized the lectin enriched sub-proteomes from these species. An attempt to characterize the modified residue and the *N*-glycan structures by MS represented the first real advancement in the area (Zhang et al., 2011). Using a combination of various glycoprotein enrichment strategies and informatics, 127 putative glycoproteins were identified by MS, with *N*-glycan sites and structures determined by prediction and re-analysis of MS1 scans (Zhang et al., 2011). Only a year later, a significant advance in the identification of *N*-glycosylation sites in plant proteins occurred as part of a large-scale survey of model eukaryotic organisms applying the developed glycoproteomic strategies (Zielinska et al., 2012). While a standard workflow of lectin affinity to enrich glycopeptides followed by treatment with peptide-*N*-glycosidase (PNGase) F was used for non-plant species, enriched plant glycopeptides from Arabidopsis were treated with PNGase A to remove the more complex fucose containing *N*-glycans. Significantly, removal of *N*-glycan structures was undertaken in the presence of H${}_{2}^{18}$O resulting in an isotopic signature on the modified residue. Thus, in combination with the deamidation of Asn to Asp by the PNGase reaction, both the site and presence of the *N*-glycan could be validated in the resultant peptides after analysis by MS. Over 2000 *N*-glycosylation sites were mapped to Arabidopsis proteins by this study, however, no structural information about the glycans was achieved (Zielinska et al., 2012). While lectin enrichment of glycans has been widely employed in glycoproteomic studies, complementary technologies have been developed including the enrichment of glycopeptides by crosslinking chemically activated carbohydrates to hydrazide beads (Zhang et al., 2003). The technique has been applied to plant samples (Arabidopsis) to both assess the specificity of PNGase (F and A) and profile *N*-glycans from wild-type and the *cgl N*-glycosylation mutant (Song et al., 2013). A total of 330 glycopeptides from 173 Arabidopsis proteins were identified using this approach. Surprisingly, it was found that the activities of commercially available PNGase A were ineffective with glycoproteomic workflows and that the identified glycopeptides were likely to have harbored immature or mannose-type glycans, like those found in the Arabidopsis *cgl* mutant (Song et al., 2013). It should be noted that this ineffective removal of complex *N*-glycans with PNGase A occurred with enriched glycopeptide fractions rather than with glycoproteins, which are known to be difficult to digest with PNGases. The focus in plant glycoproteomics has thus far mainly concentrated on the reference plant Arabidopsis with about 2500 *N*-glycan sites mapped (Mann et al., 2013). Nonetheless, the small number of glycoproteomic studies undertaken in plants and the limited species analyzed indicate that the current workflows are providing limited opportunities to study plant glycoproteomes and new or complementary approaches need to be explored.

The development of different fragmentation techniques available in the current generation of MS instruments may provide an alternative approach to the limitations of current glycoproteomic procedures (Scott et al., 2011). The most widely employed fragmentation technique in proteomic surveys to produce tandem mass spectra is collision-induced dissociation (CID). The process involves the acceleration of molecules (peptides) which are collided with a neutral gas (e.g., nitrogen) resulting in the breaking of molecular bonds and the generation of tandem mass spectra (Sleno and Volmer, 2004). The term CID generally encompasses trap-type (derived from an ion trap instrument) and beam-type [derived from a quadrupole-time-of-flight (Q-ToF) instruments]. With the development of the Orbitrap MS, a CID-based approach termed higher-energy collisional dissociation (HCD) was developed where fragmentation spectra are produced outside the iontrap, namely in the C-trap (Olsen et al., 2007). The resultant HCD fragmentation spectra are similar to those when producing CID in a Q-ToF mass spectrometer (Michalski et al., 2012). A further fragmentation technique used in proteomics is electron-transfer dissociation (ETD) or electron-capture dissociation (ECD) which causes fragmentation via the transfer of electrons to the gas phase ion (Zubarev et al., 1998; Syka et al., 2004). The application of ETD in proteomics is mainly associated with the analysis of PTMs since fragment ions tend to retain the modifications which are often lost in CID approaches (Wiesner et al., 2008). All these fragmentation techniques are now common options for most modern tandem MS.

Consequently, with the recent availability of differential MS-based fragmentation techniques, we were interested in exploring these approaches for the structural characterization of glycoproteins. The amount and purity of a specific glycoprotein is crucial for the adoption of the LC-MS^n^ approach when applying complementary fragmentation techniques. As a result, we have been using *in planta* synthesis in *Nicotiana benthamiana* to transiently express proteins with suspected *N*-glycan modifications followed by immunoprecipitation as a means to adequately enrich proteins prior to analysis by MS. Recent glycoproteomic analyses in Arabidopsis (Zielinska et al., 2012) had suggested that many enzymes found in the plant Golgi apparatus and involved in cell wall biosynthesis may themselves be glycosylated. Consequently, we have been attempting to identify *N*-glycans from recently characterized plant glycosyltransferases (Song et al., 2015) after *in planta* synthesis and immunoprecipitation. Agrobacterium harboring a construct comprising *IRREGULAR XYLEM 9* from *Asparagus* (*AoIRX9*, KJ556998) conjugated to the yellow fluorescent protein (Venus) was used to infiltrate *N. benthamiana* leaves and after 4 days, infected leaves were harvested and microsomal protein isolated. The AoIRX9 protein was enriched by immunoprecipitation with GFP-Trap® (ChromoTek) and digested with trypsin. The analysis of the digested protein lysate by standard LC-MS^n^ (CID) resulted in the identification of AoIRX9 with high sequence coverage and indicated it was considerably enriched in the lysate. The utilization of different fragmentation techniques provides complementary information to sequence a glycopeptide. For example, the utilization of ETD in combination with CID (MS^3^) has been used to produce relevant structural and sequence information from an *N*-glycopeptide (Catalina et al., 2007). The AoIRX9 digested lysate was analyzed by HCD and spectra manually inspected for the presence of an oxonium ion (204.09 m/z) likely derived from a HexNAc. A putative glycopeptide was identified with multiple charge states (770.94 *m/z* [M + 5H]^5+^, 963.43 [M + 4H]^4+^ and 1284.23 [M + 3H]^3+^) with [M + 5H]^5+^ the predominant form. The 770.94 m/z [M + 5H]^5+^ ion was subsequently analyzed using ETD to determine the peptide sequence. The three fragmentation modes, (CID, HCD, and ETD) used to characterize this enriched plant glycopeptide from AoIRX9 are outlined in Figure 1.

**Figure 1:**
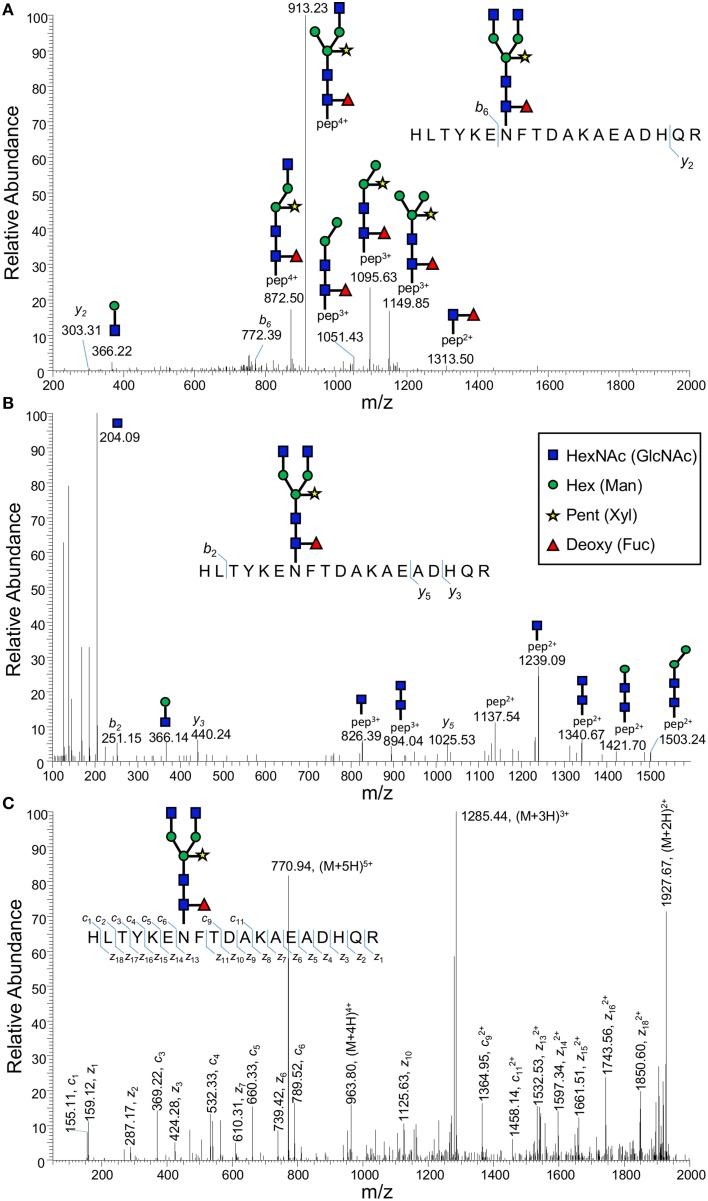
**Fragmentation spectra of a plant glycopeptide using CID, HCD, and ETD**. An immunoprecipitated protein (AoIRX9) was digested with trypsin and analyzed by LC-MS^n^ using an Orbitrap Elite™ Hybrid Ion Trap-Orbitrap (Thermo Fisher Scientific, USA). The digested sample was analyzed in triplicate using a data-dependent acquisition method incorporating a specific activation type **(A)** CID, **(B)** HCD, and **(C)** ETD. The resultant MS^n^ data (CID) were used to confirm the presence of the purified protein in the sample. Unmatched spectra from the HCD analysis were manually inspected for the presence of oxonium ions to identify putative glycopeptides in the sample. A precursor of 770.94 *m/z* [M+5H]^5+^ was identified and resultant MS^n^ spectra manually inspected to determine the mass of the peptide without the *N*-glycan. The subsequent analysis of 770.94 *m/z* [M + 5H]^5+^ by ETD revealed the sequence of the peptide. This complementary approach identified the peptide HLTYKENFTDAKAEADHQR with a complex *N*-linked glycan comprising HexNAc_4_Pent_1_Deoxy_1_Hex_3_.

The application of CID (trap-type) produced poor peptide and glycan fragmentation resulting in little peptide backbone information (*b*_6_ and *y*_2_) and glycopeptide fragments with high charge states, as previously reported (Catalina et al., 2007; Desaire, 2013). The glycan derived peaks could only be assigned after analysis of the complementary fragmentation spectra was undertaken (Figure 1A). The HCD-derived spectra (Figure 1B) revealed glycan signatures, such as the HexNAc (GlcNAc) oxonium ion (204.09 *m/z*) and importantly resulted in an ion representing the mass of the peptide without the glycan structure (1137.54 *m/z* [M + 2H]^2+^). This information is essential for the calculation of the mass of the glycan, 1576.6 Da, corresponding to a glycan structure of HexNAc_4_Pent_1_Deoxy_1_Hex_3_, which is most likely GlcNAc_4_Xyl_1_Fuc_1_Man_3_, a commonly found plant *N*-glycan (Oxley et al., 2004; Song et al., 2011). To accurately determine the structure of the glycan, a coupled approach employing detailed analysis of fragment ions and enzymatic sequencing (e.g., exoglycosidase) are necessary (Harvey, 2005). Similar to CID, the HCD spectrum provides minimal information about the peptide backbone, yielding masses for *b*_2_, *y*_3_, and *y*_5_. In contrast, the ETD spectrum (Figure 1C) was essential for fragmenting the peptide backbone revealing virtually every *z*- and a majority of *c*-ions, enabling confirmation of the peptide sequence as has been previously demonstrated for ETD (Catalina et al., 2007). Lastly, the ETD spectra enabled the determination of the residue (Asn, N) where the glycan structure was attached. In this instance, only a single likely site of *N*-glycan attachment is possible, however this situation is not always the case.

A considerable bottleneck when attempting to characterize protein glycosylation by MS is the limitations of commonly used software for high confidence matching of glycopeptide fragmentation spectra (Woodin et al., 2013). This issue becomes even more problematic when tandem spectra are derived from fragmentation techniques such as ETD. Although there is an assortment of software options ranging from commercial to open source, each has its limitations depending on the workflow (Dallas et al., 2013). Nonetheless, high throughput matching of glycopeptide spectra is still a considerable challenge which requires extensive manual curation. Generally, we have found that the presence of oxonium ions by manual inspection of HCD derived spectra is still the most reliable approach, however the identity of the glycopeptide is still required. We have attempted to analyze ETD spectra derived from glycopeptides with commonly used programs such as Mascot (Matrix Science, UK), such software is often limited in the number of modifications and the expanded search space results in significant increases in processing time. Consequently, we have found that “boutique software,” such as Byonic (Protein Metrics, USA) outperforms many of the more widely utilized programs for the analysis of ETD spectra containing a highly variable PTM. Overall, the issue of separate analyses e.g., HCD and ETD, the requirement to cross-reference data and variable modifications creates a complicated workflow which may be overcome in the future through software enhancements.

The application of complementary fragmentation techniques to characterize glycopeptides provides an effective approach in glycoproteomics (Desaire, 2013). The utilization and success of complementary fragmentation techniques for the analysis of PTMs including glycopeptides has been widely discussed (Catalina et al., 2007; Chi et al., 2007; Alley et al., 2009; Sobott et al., 2009; Snovida et al., 2010; Scott et al., 2011; Ruiz-May et al., 2012b), however the approach is yet to be adopted by the plant proteomics community. The glycopeptide example outlined here further highlights and confirms the effectiveness of HCD and ETD in revealing crucial information necessary for the characterization of both the glycan and the peptide (Scott et al., 2011). The utilization of CID is generally not of great assistance, although could contribute important structural information about the glycan from resultant fragment ions. The presence of the oxonium ion derived from the glycan in the HCD spectra (e.g., HexNAc, 204.09 *m*/*z*) should enable more advanced workflows to be employed for the characterization of glycopeptides (Wu et al., 2007; Scott et al., 2011). For example, such an application could involve the presence of the oxonium ion in the HCD fragmentation spectra triggering the ETD fragmentation of the precursor (Saba et al., 2012). A further advancement could be gained by coupling hydrophilic interaction liquid chromatography (HILIC) as the pre-MS separation procedure (Scott et al., 2011). There is a clear advantage for glycopeptide separation as features like oligosaccharide branching provide the necessary requirements for hydrophilic interactions (Zauner et al., 2011).

Finally, with advancements in hardware, it is simple to envision future instruments that simultaneously undertake a variety of fragmentation processes during a standard proteomics workflow. This improvement would also likely result in the development of software that could better deal with this complicated workflow. Such a feature could enable a global analysis of PTMs in conjunction with protein identification and label free quantification. The example outlined above applying complementary analytical approaches will likely be an essential part of future proteomic workflows for the accurate characterization of protein structure and function.

## Conflict of interest statement

The authors declare that the research was conducted in the absence of any commercial or financial relationships that could be construed as a potential conflict of interest.

## References

[B1] Alley W. R. Jr. Mechref Y. Novotny M. V. (2009). Characterization of glycopeptides by combining collision-induced dissociation and electron-transfer dissociation mass spectrometry data. Rapid Commun. Mass Spectrom. 23, 161–170. 10.1002/rcm.3850 19065542

[B2] Catalá C. Howe K. J. Hucko S. Rose J. K. Thannhauser T. W. (2011). Towards characterization of the glycoproteome of tomato (*Solanum lycopersicum*) fruit using Concanavalin A lectin affinity chromatography and LC-MALDI-MS/MS analysis. Proteomics 11, 1530–1544. 10.1002/pmic.201000424 21381198

[B3] Catalina M. I. Koeleman C. A. M. Deelder A. M. Wuhrer M. (2007). Electron transfer dissociation of *N*-glycopeptides: loss of the entire *N*-glycosylated asparagine side chain. Rapid Commun. Mass Spectrom. 21, 1053–1061. 10.1002/rcm.2929 17311219

[B4] Chi A. Huttenhower C. Geer L. Y. Coon J. J. Syka J. E. P. Bai D. L.. (2007). Analysis of phosphorylation sites on proteins from *Saccharomyces cerevisiae* by electron transfer dissociation (ETD) mass spectrometry. Proc. Natl. Acad. Sci. U.S.A. 104, 2193–2198. 10.1073/pnas.0607084104 17287358 PMC1892997

[B5] Dallas D. C. Martin W. F. Hua S. German J. B. (2013). Automated glycopeptide analysis–review of current state and future directions. Brief. Bioinformatics 14, 361–374. 10.1093/bib/bbs045 22843980 PMC3659302

[B6] Desaire H. (2013). Glycopeptide analysis, recent developments and applications. Mol. Cell. Proteomics 12, 893–901. 10.1074/mcp.R112.026567 23389047 PMC3617336

[B7] Furuichi T. Kayserili H. Hiraoka S. Nishimura G. Ohashi H. Alanay Y. (2009). Identification of loss-of-function mutations of SLC35D1 in patients with Schneckenbecken dysplasia, but not with other severe spondylodysplastic dysplasias group diseases. J. Med. Genet. 46, 562–568. 10.1136/jmg.2008.065201 19508970 PMC4144354

[B8] Gomord V. Fitchette A. C. Menu-Bouaouiche L. Saint-Jore-Dupas C. Plasson C. Michaud D.. (2010). Plant-specific glycosylation patterns in the context of therapeutic protein production. Plant Biotechnol. J. 8, 564–587. 10.1111/j.1467-7652.2009.00497.x 20233335

[B9] Harvey D. J. (2005). Proteomic analysis of glycosylation: structural determination of *N*- and *O*-linked glycans by mass spectrometry. Expert Rev. Proteomics 2, 87–101. 10.1586/14789450.2.1.87 15966855

[B10] Heazlewood J. L. (2011). The green proteome: challenges in plant proteomics. Front. Plant Sci. 2:6. 10.3389/fpls.2011.00006 22639573 PMC3355608

[B11] Hijazi M. Durand J. Pichereaux C. Pont F. Jamet E. Albenne C. (2012). Characterization of the arabinogalactan protein 31 (AGP31) of *Arabidopsis thaliana*: new advances on the Hyp-O-glycosylation of the Pro-rich domain. J. Biol. Chem. 287, 9623–9632. 10.1074/jbc.M111.247874 22270363 PMC3308734

[B12] Kimura Y. Hess D. Sturm A. (1999). The *N*-glycans of jack bean alpha-mannosidase - Structure, topology and function. Eur. J. Biochem. 264, 168–175. 10.1046/j.1432-1327.1999.00598.x 10447685

[B13] Lerouxel O. Mouille G. Andème-Onzighi C. Bruyant M. P. Séveno M. Loutelier-Bourhis C.. (2005). Mutants in DEFECTIVE GLYCOSYLATION, an Arabidopsis homolog of an oligosaccharyltransferase complex subunit, show protein underglycosylation and defects in cell differentiation and growth. Plant J. 42, 455–468. 10.1111/j.1365-313X.2005.02392.x 15860005

[B14] Lige B. Ma S. Van Huystee R. B. (2001). The effects of the site-directed removal of *N*-glycosylation from cationic peanut peroxidase on its function. Arch. Biochem. Biophys. 386, 17–24. 10.1006/abbi.2000.2187 11360996

[B15] Mann G. W. Calley P. C. Joshi H. J. Heazlewood J. L. (2013). MASCP Gator: an overview of the Arabidopsis proteomic aggregation portal. Front. Plant Sci. 4:411. 10.3389/fpls.2013.00411 24167507 PMC3806167

[B16] Michalski A. Neuhauser N. Cox J. Mann M. (2012). A systematic investigation into the nature of tryptic HCD spectra. J. Proteome Res. 11, 5479–5491. 10.1021/pr3007045 22998608

[B17] Minic Z. Jamet E. Négroni L. Der Garabedian P. A. Zivy M. Jouanin L. (2007). A sub-proteome of *Arabidopsis thaliana* mature stems trapped on Concanavalin A is enriched in cell wall glycoside hydrolases. J. Exp. Bot. 58, 2503–2512. 10.1093/jxb/erm082 17526915 PMC2394711

[B18] Morelle W. Michalski J. C. (2007). Analysis of protein glycosylation by mass spectrometry. Nat. Protoc. 2, 1585–1602. 10.1038/nprot.2007.227 17585300

[B19] Nguema-Ona E. Vicré-Gibouin M. Gotté M. Plancot B. Lerouge P. Bardor M.. (2014). Cell wall *O*-glycoproteins and N-glycoproteins: aspects of biosynthesis and function. Front. Plant Sci. 5:499. 10.3389/fpls.2014.00499 25324850 PMC4183102

[B20] Olsen J. V. Macek B. Lange O. Makarov A. Horning S. Mann M. (2007). Higher-energy C-trap dissociation for peptide modification analysis. Nat. Methods 4, 709–712. 10.1038/nmeth1060 17721543

[B21] Oxley D. Currie G. C. Bacic A. (2004). Analysis of carbohydrate from glycoproteins, in Purifying Proteins for Proteomics: A Laboratory Manual, ed Simpson R. J. (New York, NY: Cold Spring Harbor Laboratory Press), 579–636.

[B22] Rayon C. Lerouge P. Faye L. (1998). The protein *N*-glycosylation in plants. J. Exp. Bot. 49, 1463–1472. 10.1093/jxb/49.326.1463

[B23] Reddy V. A. Johnson R. S. Biemann K. Williams R. S. Ziegler F. D. Trimble R. B.. (1988). Characterization of the glycosylation sites in yeast external invertase 1. *N*-linked oligosaccharide content of the individual sequons. J. Biol. Chem. 263, 6978–6985. 3284881

[B24] Rudd P. M. Dwek R. A. (1997). Glycosylation: Heterogeneity and the 3D structure of proteins. Crit. Rev. Biochem. Mol. 32, 1–100. 10.3109/10409239709085144 9063619

[B25] Ruiz-May E. Kim S. J. Brandizzi F. Rose J. K. C. (2012a). The secreted plant *N*-glycoproteome and associated secretory pathways. Front. Plant Sci. 3:117. 10.3389/fpls.2012.00117 22685447 PMC3368311

[B26] Ruiz-May E. Thannhauser T. W. Zhang S. Rose J. K. C. (2012b). Analytical technologies for identification and characterization of the plant *N*-glycoproteome. Front. Plant Sci. 3:150. 10.3389/fpls.2012.00150 22783270 PMC3389394

[B27] Saba J. Dutta S. Hemenway E. Viner R. (2012). Increasing the productivity of glycopeptides analysis by using higher-energy collision dissociation-accurate mass-product-dependent electron transfer dissociation. Int. J. Proteomics 2012:560391. 10.1155/2012/560391 22701174 PMC3369405

[B28] Scott N. E. Parker B. L. Connolly A. M. Paulech J. Edwards A. V. G. Crossett B. (2011). Simultaneous glycan-peptide characterization using hydrophilic interaction chromatography and parallel fragmentation by CID, higher energy collisional dissociation, and electron transfer dissociation MS applied to the *N*-linked glycoproteome of *Campylobacter jejuni*. Mol. Cell. Proteomics 10:M000031-MCP201. 10.1074/mcp.M000031-MCP201 20360033 PMC3033663

[B29] Sleno L. Volmer D. A. (2004). Ion activation methods for tandem mass spectrometry. J. Mass Spectrom. 39, 1091–1112. 10.1002/jms.703 15481084

[B30] Snovida S. I. Bodnar E. D. Viner R. Saba J. Perreault H. (2010). A simple cellulose column procedure for selective enrichment of glycopeptides and characterization by nano LC coupled with electron-transfer and high-energy collisional-dissociation tandem mass spectrometry. Carbohydr. Res. 345, 792–801. 10.1016/j.carres.2010.01.006 20189550

[B31] Sobott F. Watt S. J. Smith J. Edelmann M. J. Kramer H. B. Kessler B. M. (2009). Comparison of CID versus ETD based MS/MS fragmentation for the analysis of protein ubiquitination. J. Am. Soc. Mass Spectrom. 20, 1652–1659. 10.1016/j.jasms.2009.04.023 19523847

[B32] Song L. Zeng W. Wu A. Picard K. Lampugnani E. R. Cheetamun R.. (2015). Asparagus spears as a model to study heteroxylan biosynthesis during secondary wall development. PLoS ONE 10:e0123878. 10.1371/journal.pone.0123878 25894575 PMC4404143

[B33] Song W. Henquet M. G. L. Mentink R. A. Van Dijk A. J. Cordewener J. H. G. Bosch D.. (2011). *N*-glycoproteomics in plants: perspectives and challenges. J. Proteomics 74, 1463–1474. 10.1016/j.jprot.2011.05.007 21605711

[B34] Song W. Mentink R. A. Henquet M. G. L. Cordewener J. H. G. Van Dijk A. D. J. Bosch D.. (2013). *N*-glycan occupancy of Arabidopsis *N*-glycoproteins. J. Proteomics 93, 343–355. 10.1016/j.jprot.2013.07.032 23994444

[B35] Strasser R. (2014). Biological significance of complex N-glycans in plants and their impact on plant physiology. Front. Plant Sci. 5:363. 10.3389/fpls.2014.00363 25101107 PMC4105690

[B36] Syka J. E. Coon J. J. Schroeder M. J. Shabanowitz J. Hunt D. F. (2004). Peptide and protein sequence analysis by electron transfer dissociation mass spectrometry. Proc. Natl. Acad. Sci. U.S.A. 101, 9528–9533. 10.1073/pnas.0402700101 15210983 PMC470779

[B37] Tretter V. Altmann F. Marz L. (1991). Peptide-*N*^4^-(*N*-acetyl-b-glucosaminyl)asparagine amidase-F cannot release glycans with fucose attached a1-3 to the asparagine-linked *N*-acetylglucosamine residue. Eur. J. Biochem. 199, 647–652. 10.1111/j.1432-1033.1991.tb16166.x 1868849

[B38] Van den Steen P. Rudd P. M. Dwek R. A. Opdenakker G. (1998). Concepts and principles of *O*-linked glycosylation. Crit. Rev. Biochem. Mol. Biol. 33, 151–208. 10.1080/10409239891204198 9673446

[B39] Welinder K. G. Tams J. W. (1995). Effects of glycosylation on protein folding, stability and solubility. Studies of chemically modified or engineered plant and fungal peroxidases. Progr. Biotechnol. 10, 205–210. 10.1016/S0921-0423(06)80104-8

[B40] Wiesner J. Premsler T. Sickmann A. (2008). Application of electron transfer dissociation (ETD) for the analysis of posttranslational modifications. Proteomics 8, 4466–4483. 10.1002/pmic.200800329 18972526

[B41] Woodin C. L. Maxon M. Desaire H. (2013). Software for automated interpretation of mass spectrometry data from glycans and glycopeptides. Analyst 138, 2793–2803. 10.1039/c2an36042j 23293784 PMC3633625

[B42] Wu S. L. Hühmer A. F. Hao Z. Karger B. L. (2007). On-line LC-MS approach combining collision-induced dissociation (CID), electron-transfer dissociation (ETD), and CID of an isolated charge-reduced species for the trace-level characterization of proteins with post-translational modifications. J. Proteome Res. 6, 4230–4244. 10.1021/pr070313u 17900180 PMC2557440

[B43] Zauner G. Deelder A. M. Wuhrer M. (2011). Recent advances in hydrophilic interaction liquid chromatography (HILIC) for structural glycomics. Electrophoresis 32, 3456–3466. 10.1002/elps.201100247 22180202

[B44] Zhang H. Aebersold R. (2006). Isolation of glycoproteins and identification of their *N*-linked glycosylation sites. Methods Mol. Biol. 328, 177–185. 10.1385/1-59745-026-X:177 16785649

[B45] Zhang H. Li X. J. Martin D. B. Aebersold R. (2003). Identification and quantification of *N*-linked glycoproteins using hydrazide chemistry, stable isotope labeling and mass spectrometry. Nat. Biotechnol. 21, 660–666. 10.1038/nbt827 12754519

[B46] Zhang Y. Giboulot A. Zivy M. Valot B. Jamet E. Albenne C. (2011). Combining various strategies to increase the coverage of the plant cell wall glycoproteome. Phytochemistry 72, 1109–1123. 10.1016/j.phytochem.2010.10.019 21109274

[B47] Zhou Q. Kyazike J. Echelard Y. Meade H. M. Higgins E. Cole E. S.. (2005). Effect of genetic background on glycosylation heterogeneity in human antithrombin produced in the mammary gland of transgenic goats. J. Biotechnol. 117, 57–72. 10.1016/j.jbiotec.2005.01.001 15831248

[B48] Zhou Y. Aebersold R. Zhang H. (2007). Isolation of N-linked glycopeptides from plasma. Anal. Chem. 79, 5826–5837. 10.1021/ac0623181 17591751

[B49] Zielinska D. F. Gnad F. Schropp K. Wisniewski J. R. Mann M. (2012). Mapping *N*-glycosylation sites across seven evolutionarily distant species reveals a divergent substrate proteome despite a common core machinery. Mol. Cell 46, 542–548. 10.1016/j.molcel.2012.04.031 22633491

[B50] Zubarev R. A. Kelleher N. L. McLafferty F. W. (1998). Electron capture dissociation of multiply charged protein cations. A nonergodic process. J. Am. Chem. Soc. 120, 3265–3266. 10.1021/ja973478k

